# Helium Generated Cold Plasma Finely Regulates Activation of Human Fibroblast-Like Primary Cells

**DOI:** 10.1371/journal.pone.0104397

**Published:** 2014-08-15

**Authors:** Paola Brun, Surajit Pathak, Ignazio Castagliuolo, Giorgio Palù, Paola Brun, Matteo Zuin, Roberto Cavazzana, Emilio Martines

**Affiliations:** 1 Department of Molecular Medicine, Unit of Microbiology, University of Padova, Padova, Italy; 2 Department of Surgical, Oncological and Gastroenterological Sciences, University of Padova, Padova, Italy; 3 Department of Molecular Medicine, Unit of Histology, University of Padova, Padova, Italy; 4 Consorzio RFX, Padova, Italy; 5 Istituto Gas Ionizzati del CNR, Padova, Italy; University Paul Sabatier, France

## Abstract

Non-thermal atmospheric pressure plasmas are being developed for a wide range of health care applications, including wound healing. However in order to exploit the potential of plasma for clinical applications, the understanding of the mechanisms involved in plasma-induced activation of fibroblasts, the cells active in the healing process, is mandatory. In this study, the role of helium generated plasma in the tissue repairing process was investigated in cultured human fibroblast-like primary cells, and specifically in hepatic stellate cells and intestinal subepithelial myofibroblasts. Five minutes after treatment, plasma induced formation of reactive oxygen species (ROS) in cultured cells, as assessed by flow cytometric analysis of fluorescence-activated 2′,7′-dichlorofluorescein diacetate probe. Plasma-induced intracellular ROS were characterized by lower concentrations and shorter half-lives with respect to hydrogen peroxide-induced ROS. Moreover ROS generated by plasma treatment increased the expression of peroxisome proliferator activated receptor (PPAR)-γ, nuclear receptor that modulates the inflammatory responses. Plasma exposure promoted wound healing in an *in vitro* model and induced fibroblast migration and proliferation, as demonstrated, respectively, by trans-well assay and partitioning between daughter cells of carboxyfluorescein diacetate succinimidyl ester fluorescent dye. Plasma-induced fibroblast migration and proliferation were found to be ROS-dependent as cellular incubation with antioxidant agents (e.g. *N*-acetyl L-cysteine) cancelled the biological effects. This study provides evidence that helium generated plasma promotes proliferation and migration in liver and intestinal fibroblast-like primary cells mainly by increasing intracellular ROS levels. Since plasma-evoked ROS are time-restricted and elicit the PPAR-γ anti-inflammatory molecular pathway, this strategy ensures precise regulation of human fibroblast activation and can be considered a valid therapeutic approach for liver and gut lesions.

## Introduction

Non-thermal atmospheric-pressure plasmas have recently been investigated for multiple medical applications [1]. Besides sterilizing inert surfaces [2], disinfecting living tissue [3], [4], disposing of cancer cells and bleeding control [5]–[7], cold plasmas of various gas compositions have been found to promote wound healing and tissue regeneration [8]–[10]. Nevertheless plasma functionalization of medical implant devices enhances the attachment and spreading of cells *in vivo*, suggesting that plasma can also interact with the extracellular environment shaping the properties of surface proteins [11], [12]. Most of plasma's effects are strictly linked to reactive oxygen species (ROS) production [3], [5]. Indeed, ROS generated during plasma exposure could be responsible for lipid peroxidation of membrane in bacteria [13], releasing growth factors in proliferating cells [14], and DNA damage in cancer cell lines [6], [15]. In the gas phase, non-thermal plasmas contain neutral non-charged species which, following interaction with organic cellular components as well as media enriched in specific amino acids, lead to the production of excited molecules, ions, and radicals [16]. This is why some concerns have been expressed with regard to employing plasma in *in vivo* medical treatments and in wound healing. For example, non-thermal atmospheric pressure plasma affects the expression of adhesion molecules on cellular surfaces causing loss of cell-cell interaction and cellular detachment in HaCaT-keratinocytes [17]. Despite a large body of scientific evidence that has been collected with regard to immortalized cell lines, few studies have been performed until now to investigate the effects of plasmas on cell adhesion and proliferation of primary fibroblasts [18], the main cell population contributing to the healing process.

Wound healing is a dynamic, well-organized process requiring the cooperation of different cell types to repair skin lesions and damaged internal organs [19]. At first, low local levels of cytokines, chemokines, and components of the renin-angiotensin system trigger proliferation and migration of mesenchymal and non-mesenchymal cells to promote wound healing. But the critical event in wound healing is the transition of tissue resident cells to activated fibroblasts which produce growth factors and a collagen framework that promote tissue regeneration [20], [21]. One of several mediators, ROS regulate myofibroblast activation and play a key role in extracellular matrix deposition [22]. Thus, upon toxic insult or viral infection, reactive oxygen intermediates are mandatory to activate and differentiate quiescent hepatic stellate cells (HSCs) into contractile myofibroblast-like cells, the main source of extracellular matrix components during wound healing responses in the liver [23]. Threshold levels of ROS are likewise critical in maintaining epithelial restitution, reconstitution, and barrier function in intestinal subepithelial myofibroblasts (ISEMFs), whereas increased ROS levels have been linked to gut fibrosis [24]. The physiological wound healing process is, however, often jeopardized by intrinsic and environmental factors. Indeed, infiltrating leukocytes, neighbouring damaged cells, and/or microbes infecting the wound increase inflammatory cytokine levels and exaggerate ROS generation thus perpetuating myofibroblast activation with excessive accumulation of collagen and fibrotic scarring [25], [26].

We recently reported that atmospheric pressure non-thermal plasma needle generated by ionizing helium gas slightly increased intracellular ROS levels in primary human keratocytes and conjunctival fibroblasts [3]. The aim of the present study was to investigate the effects of plasma-induced ROS on the proliferation and migration of cultured human primary HSCs and ISEMFs and to specifically assess if controlling myofibroblast activation and modulating their pro-fibrogenic effects can accelerate the wound healing without fibrotic scar formation.

## Materials and Methods

### Cell culture

Human HSCs were freshly isolated from non-pathological fragments of liver tissue collected from 3 patients during surgical resection of liver metastases. The samples were processed separately and HSCs were cultured as described elsewhere [27]. Briefly, following digestion with collagenase and pronase, HSCs were isolated by centrifugation over a gradient of Percoll (Amersham Biosciences, Sweden) and cultured in DMEM containing 10% vol/vol fetal bovine serum (FBS), 2 mM L-glutamine, 100 U/ml penicillin, and 100 µg/ml streptomycin, (all provided by Gibco, Italy). The purity of cultured HSCs was assessed by immunocytochemistry using anti-αSMA antibody (Sigma, Italy).

ISEMFs were isolated from non-pathological colonic biopsies collected from 3 subjects undergoing colonoscopy for cancer screening. The tissue samples were diced, extensively washed, and digested for 30 min at 37°C in collagenase (0.25 mg/ml, Sigma). Recovered cells were suspended in DMEM with 20% vol/vol FBS, 2 mM L-glutamine, 1 mM sodium pyruvate, 0.1 mM nonessential amino acids, 100 U/ml penicillin, 100 µg/ml streptomycin and 2 ng/mL fungizone (Gibco). The purity of the cultured ISEMFs was ascertained by fluorescence-activated cell sorting (FACS) analysis using anti-CD90 antibody (ImmunoTools, Germany). Primary cells were maintained at 37°C in a 5% CO_2_ humidified incubator. At confluence, the cells were detached using 0.05% Trypsin-EDTA (Gibco).

The study protocol was designed in accordance with the principles expressed in the Declaration of Helsinki and was approved by the Ethical Committee of the University Hospital of Padova. Patients were provided with detailed information about the study aims and protocol and gave their written, informed consent.

### Atmospheric pressure cold plasma

Cultured cells were exposed to the effluent coming from a plasma (ionized gas) source specifically designed to treat living tissues. The plasma was produced by applying a radiofrequency (RF) electric field to a flow of helium at atmospheric pressure. The source operated at a very low power level so that the resulting plasma was characterized by a low fraction of ionized particles (ions and electrons). The neutral gas fraction and the ion flow were at room temperature. The electron population was at a temperature to the order of 1 eV (11600 K). Generally called “cold plasma,” this plasma source is considered suitable for treating biological samples without thermal effects. The interaction of the electron population with the surrounding air mixed to the helium flow stimulates the production of active chemicals such as ROS, atomic oxygen and hydroxyl radical, and probably accounts for its biological effects.

**Figure 1:**
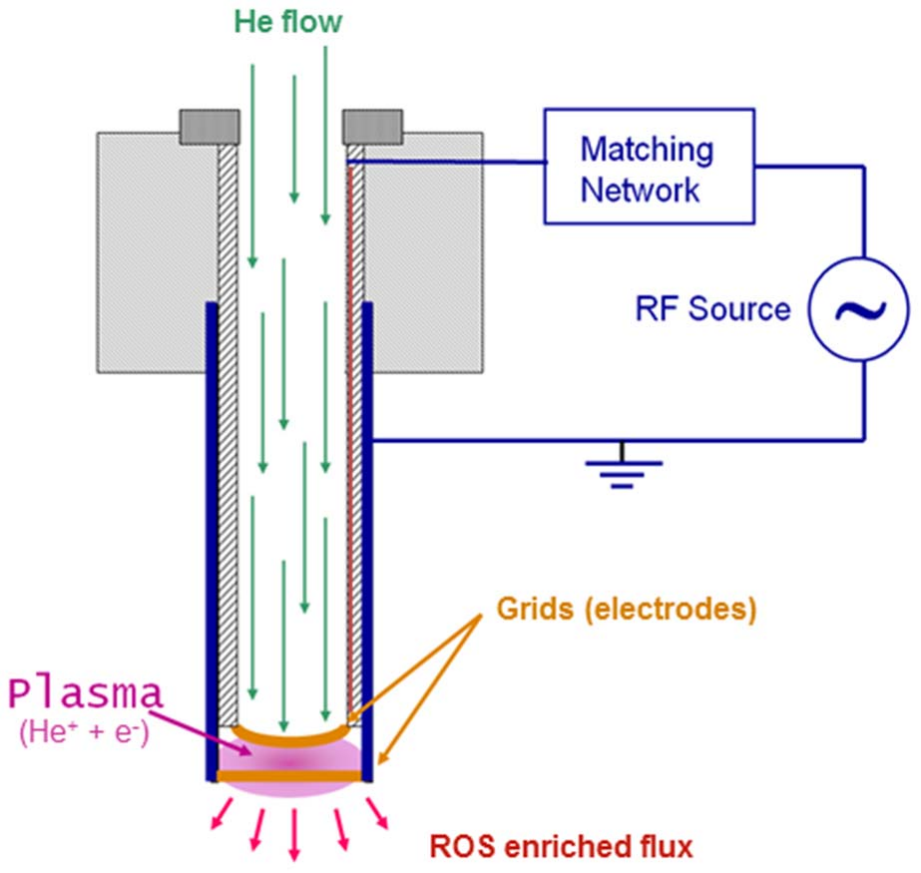
Schematic representation of helium generated plasma source. The plasma source used in the present study consisted of two coaxial tubes, the outer one made out of copper and the inner one made out of insulating material. Two parallel grids 1 mm away from one another closed the tubes. The electric field was formed in the space between the two grids by applying a RF voltage difference supplied by a radiofrequency (RF) generator coupled to the source by a matching network.

Plasma was generated between two grids acting as electrodes, and the cultured cells were exposed to the so-called afterglow, which is the chemical-enriched helium flow. In contrast to other cold plasma devices used in biomedical applications, the plasma-induced charged species did not have a direct biological effect on the cultured cells since they recombined very quickly before reaching the samples. This plasma source has been described elsewhere [28] and is schematically outlined in Figure 1. As indicated, the source consists of two coaxial tubes, each closed at one end by a double brass grid. The outer tube is made out of copper and electrically grounded while the inner one is made out of insulating material. The two parallel grids are positioned 1 mm away from one other. An electric field is formed in the space between the two grids by applying a RF voltage difference supplied by a RF generator coupled to the source by a matching network. The matching network raises the voltage to the value needed for helium ionization of about 1000 V peak-to-peak. Despite the high voltage value, the current flowing in the plasma is so low that the dissipated power is below 1 W. The chosen operational frequency is 4.8 MHz. The gas flow rate is 1.5 litres/min.

### Detection of intracellular ROS levels

HSCs and ISEMFs were seeded in 24-well tissue culture plates (Corning, Italy) at 3×10^5^ cells/ml to assess ROS generation. Twenty-four hrs later cells were loaded for 30 min at 37°C with 10 µM 2′,7′-dichlorodihydrofluorescein diacetate (H_2_DCFDA; Molecular Probes, Invitrogen, Italy) in warm PBS [3]. At the end of the incubation, cultures were washed twice, incubated with 200 µl of fresh serum-free culture medium, and exposed for 1, 2, 5, or 10 min to atmospheric pressure cold plasma. The tip of the plasma source was positioned within 1.50 to 2.40 mm from the surface of the medium soaking the samples. The cells were then incubated at 37°C for 5 min–24 hrs, harvested by Trypsin-EDTA, washed and analyzed using BD FACS-Calibur flow cytometer (Becton Dickinson, USA). Ten thousands events were acquired for each sample. The results were analyzed using the WinMDI 2.9 (Windows Multiple Document Interface for Flow Cytometry) program. In a different set of experiments, the cells were treated for 15 minutes at 37°C with 5 mM *N*-acetyl L-cysteine (NAC), 20 µM glutathione (GSH), 50 µM α-tocopherol, 1000 U/ml catalase or 20 µM H_2_O_2_ (all provided by Sigma), all conditions previously established in our laboratories.

### Mitochondrial dysfunction

Plasma treated cells or cells stimulated with H_2_O_2_ were incubated for 1, 6, 24, 48 hrs at 37°C. At the end of the incubation, the cells were collected and re-suspended in PBS containing 1 µM JC-1 (5,5′,6,6′-tetrachloro-1,1′,3,3′-tetraethylbenzimidazol-carbocyanine, Sigma). The cytofluorimetric analysis was performed collecting green and orange fluorescence for JC-1 staining in 10,000 events for each sample.

### Evaluation of lipid peroxidation

Lipid peroxidation was determined by measuring the thiobarbituric acid (TBA, Inalco, Italy) reactive substances (TBA-reactants). Plasma treated HSCs and ISEMFs were incubated for 5 min or 6 hrs. At the end of the incubation, 20% w/vol cold trichloroacetic acid was added to cells and centrifuged. Supernatants were incubated with 0.67% solution of TBA at 100°C for 10 min. The absorbance was recorded at 532 nm. Values were plotted on a standard curve obtained by serial dilution of malonaldehyde tetrabutylammanium salt (Sigma) and the TBA-reactant level was calculated using a molar extinction coefficient of 1.56×10^5^M^−1^×cm^−1^. The results for each sample were normalized to the protein concentration determined by bicinchoninic acid assay (Pierce, Italy).

### Cell death assay

Apoptotic loss of plasma membrane asymmetry or necrotic loss of plasma membrane integrity were assessed at 1, 6, 12, 24, and 48 hrs after plasma treatment by labeling cells with Annexin V–FITC and propidium iodide (PI), according to the manufacturer's instructions (Annexin V Fluos, Roche Diagnostic, Italy). Samples were incubated in the dark for 15 min and then analyzed using a BD FACS-Calibur flow cytometer. Ten thousands events were acquired for each sample.

### Western blot analysis

Plasma treated HSCs and ISEMFs were cultured for 4 hrs. The cells were then washed with ice-cold PBS and subjected to total protein extraction in no-denaturing RIPA buffer, as described elsewhere [27]. Forty µg of proteins was separated for each sample in 7% w/vol SDS-PAGE and finally transferred to PVDF membrane (BioRad, Italy). Membranes were probed with anti-rabbit peroxisome proliferator activated receptor (PPAR)-γ antibody at 4°C for 16 hrs and then incubated with horseradish peroxidase (HRP)-conjugated secondary antibody. Anti-β-actin antibody (Sigma) was used as the loading control. Immune-complexes were visualized using enhanced chemiluminescence (ECL, Millipore). Images were captured using a Hyper film MP (Sigma).

### Cytokine ELISA

HSCs and ISEMFs were incubated for 30 min with PPAR-γ receptor antagonist T0070907 (50 µM, Tocris, UK) or with vehicle alone. Interleukin (IL)-1 and IL-6, tumour necrosis factor (TNF)-α and transforming growth factor (TGF)-β1 were assessed in the conditioned medium 24 hrs after plasma treatment using commercially available ELISA kits (e-Bioscience, Prodotti Gianni, Italy). Optical densities were measured at 450 nm using a micro plate reader (Sunrise, Tecan; Switzerland). The sensitivity of the assays ranged between 1 and 15 pg/ml.

### Proliferation assay

Cells (3×10^5^/ml) were incubated at 37°C for 10 min in pre-warmed PBS containing 0.1% vol/vol BSA (Sigma) and 25 µM carboxyfluorescein diacetate succinimidyl ester (CFSE, Molecular Probe, Invitrogen). Staining was quenched by adding 5 volumes of ice-cold culture media. Sixteen hrs later the cells were washed, counted using Trypan blue, seeded at 8×10^4^ cells/ml, and treated with NAC, as described elsewhere, or with 5 µg/ml cytochalasin B (Sigma). Cultures were then exposed to plasma and subsequently incubated in fresh culture medium at 37°C for 72 hrs. Cell proliferation was evaluated by the partitioning of fluorescent dye between daughter cells using BD FACS-Calibur flow cytometer.

### Migration assay

The cells were labelled for 10 min at 37°C with 5 µM 6-carboxyfluorescein diacetate (6-CFDA, Molecular Probe, Invitrogen) cell-permeable esterase substrate and exposed to plasma. The cells were then seeded (8×10^4^ cells/ml) in DMEM supplemented with 0.2% vol/vol FBS into trans-well inserts (8 µm pore size, Corning, the Netherlands), held in 12-well tissue culture plates containing complete culture medium and cultured for 72 hrs. The bottom side of the trans-well inserts was washed twice and the cells were harvested using cell scrapers. The cell membrane was dissolved in PBS containing 0.5% vol/vol Triton X-100. The samples were then centrifuged at 4°C 13,000 rpm for 10 min. Fluorescence was detected in the supernatant at excitation and emission wavelengths of 485 and 530 nm, respectively (Hitachi F2000 Fluorescence Spectrophotometer, Japan).

### Wound healing assay

Plasma treated cells were seeded onto glass coverslips and cultured at 37°C for 24 hrs. The cell monolayer was then wounded with a plastic tip. Seventy-two hrs later, the cells were rinsed twice in PBS, fixed in paraformaldehyde (PFA) 4% w/vol for 10 min, and routinely stained with haematoxylin and eosin. Finally, the slides were examined using a light transmission microscope connected to a camera to capture the images (DMLB Leica, Wetzlar, Germany).

### Statistical analyses

Experiments were performed using HSCs freshly isolated from the liver specimens of 3 different subjects and ISEMFs freshly prepared from the colonic biopsies collected from 3 different subjects. Experiments were carried out two or three times in duplicate or triplicate. The graphics express data as mean ± standard error (SEM). The unpaired Student's *t*-test was used to compare the data of two groups and one-way ANOVA test followed by the Newman-Keuls post-hoc test was used to compare the data of three or more groups. Statistical analysis was performed using GraphPad Prism 3.03 (San Diego, California, USA).

## Results

### The gap between the plasma source tip and the sample affected intracellular ROS generation

**Figure 2:**
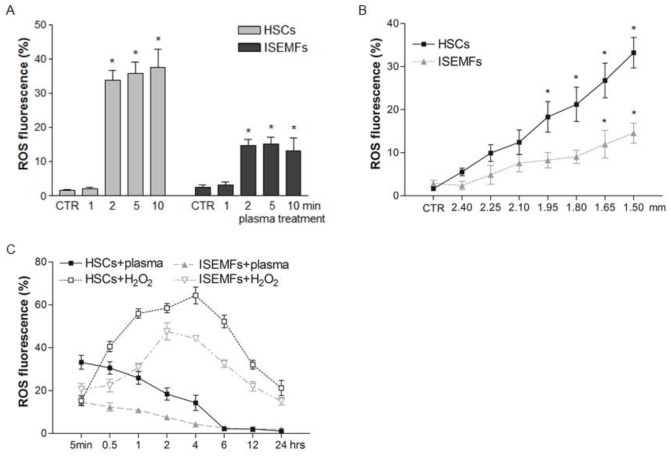
Plasma-induced intracellular ROS levels. Primary cultured human hepatic stellate cells (HSCs) and intestinal subepithelial myofibroblasts (ISEMFs) were loaded with H_2_DCFDA and exposed for 1, 2, 5, or 10 min to helium-generated plasma. The cells were cultured for 5 min under optimal conditions and then evaluated for intracellular ROS generation by FACS analysis. ROS formation was expressed as the percentage of fluorescence relative to intracellular ROS (ROS fluorescence %, panel **A**). Intracellular ROS generation was evaluated in fibroblast-like primary cells exposed for 2 min at different distances from the plasma source tip (panel **B**). The gap between the plasma source tip and the sample was fixed at 1.5 mm, and intracellular ROS generation was evaluated at different times of incubation after 2 minutes of plasma treatment or following exposure to 20 µM H_2_O_2_ (panel **C**). During FACS analysis 10,000 events were collected. Data are reported as mean±SE of the percentage of fluorescence registered in nine independent experiments, each performed in duplicate or in triplicate. * denotes P<0.05 *vs* respective not treated cells (CTR).

Several studies have reported that plasma effects are primarily due to ROS generation [3], [29]. A number of parameters such as the gas mixture, the plasma power applied, the length of exposure, the composition of the medium embedding the sample, and the distance from the plasma source tip actually affects ROS generation and in the end plasma performance [13], [15]. In the attempt to evaluate the experimental parameters influencing intracellular ROS generation in this study fibroblast-like primary cells were exposed to plasma for 1, 2, 5, and 10 min. As outlined in Figure 2A, 2 min of plasma treatment significantly increased ROS levels in both HSCs and ISEMFs. Plasma exposure for 1 min did not affect the generation of intracellular ROS, but at longer treatment times (e.g. 5 and 10 min) there was no further increase in intracellular ROS levels with respect to that following 2 min of treatment. In addition, the distance between the plasma source tip and the sample had an important effect on ROS generation. In order to assess this variable fibroblast-like primary cells were exposed for 2 min to plasma at different distances from the source tip. As demonstrated in Figure 2B, intracellular ROS levels were strongly influenced in both HSCs and ISEMFs by the distance from the plasma source tip and they peaked when the gap was fixed at 1.5 mm from the surface of the medium soaking the samples. This was the minimal distance we registered to avoid contact with the medium surface. Subsequent experiments were thus performed at this distance for 2 min of plasma exposure. Under these conditions intracellular ROS formation was not evident when the cells were exposed to the electric field without plasma, thus ruling out the role of the electric field alone in ROS formation (data not shown).

As outlined elsewhere with regard to other primary cell populations [3], plasma-generated ROS increased 5 min after plasma treatment in both HSCs and ISEMFs (Figure 2C). Plasma-generated ROS were characterized by short half-lives with respect to ROS induced in cells exposed to H_2_O_2_. In HSCs, ROS were extinguished within 6 hrs following plasma treatment while significant levels of enhanced H_2_DCFDA positive cells were still detectable even 24 hrs after H_2_O_2_ challenge. Similar results were obtained in ISEMFs (Figure 2C).

### Plasma exposure increased ROS level in cytoplasmic compartment

**Figure 3:**
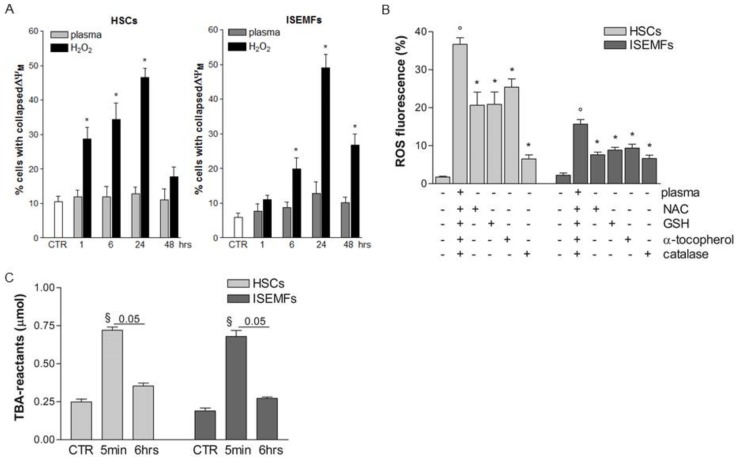
Sources of ROS in plasma-treated fibroblast-like primary cells. HSCs and ISEMFs were loaded with JC-1 and exposed to plasma or to 20 µM H_2_O_2_. Cells were cultured for 1, 6, 24 and 48 hrs under optimal conditions and mitochondrial dysfunction was evaluated by FACS analysis in 10,000 events (panel **A**). H_2_DCFDA-loaded cells were treated for 15 min with 5 mM *N*-acetyl L-cysteine (NAC), 20 µM reduced glutathione (GSH), 50 µM α-tocopherol, 1000 U/ml catalase and exposed for 2 min to plasma. After 5 min, the percentage of fluorescence relative to intracellular ROS was evaluated by FACS analysis in 10,000 events (panel **B**). Cells were treated with plasma for 2 min and incubated under standard conditions for 5 min or 6 hrs. Thiobarbituric acid (TBA)-reactants were evaluated in cell lysates and normalized to protein contents (panel **C**). Data are reported as mean±SE of results collected in nine independent experiments, each performed in duplicate or triplicate. ° denotes P<0.02 *vs* non treated cells. ^§^ denotes P<0.05 *vs* non treated cells (CTR). * denotes P<0.05 *vs* plasma-treated cells.

In the attempt to investigate mitochondrial involvement in intracellular ROS generation fibroblast-like primary cells were loaded with JC-1, a dye switching from orange fluorescent aggregates under high mitochondrial potential to green fluorescent monomeric forms after depolarization of the mitochondrial membrane [30]. As outlined in Figure 3A, incubation with H_2_O_2_ considerably increased the percentage of HSCs and ISEMFs with collapsed membrane potential at almost all the time points tested. On the contrary, plasma treatment did not significantly affect mitochondrial depolarization in the fibroblast-like primary cells, and this suggests that mitochondria are not involved in plasma-induced ROS generation.

Plasma-induced intracellular ROS levels were, however, dampened by cellular pre-treatment with different anti-oxidant agents. Thus, as shown in Figure 3B, plasma-elicited ROS generation was significantly blocked by NAC, GSH, and α-tocopherol (P<0.05 *vs* plasma-treated cells), all ROS scavengers largely used in clinical therapy, that are able to increase the cytoplasmic antioxidant pool [31]. Moreover, ROS formation was mainly prevented in HSCs by catalase indicating that hydrogen peroxide is a critical plasma by-product. Taken together, these data suggest that ROS were generated in the cytoplasm of plasma-exposed primary cells *via* oxidation of sulphide groups and/or lipids. Indeed, TBA-reactive substances, a marker of lipid peroxidation, increased in the cytoplasmic compartment of both HSCs and ISEMFs 5 min after plasma treatment but disappeared following 6 hrs of incubation (Figure 3C), a time course matching the kinetics of ROS levels (Figure 2C).

Plasma treatment, however, did not result in cellular death. Thus even after a long incubation period (48 hrs) following plasma treatment, the percentages of Annexin V positive cells as well as Annexin V and PI double positive cells did not significantly increase in HSCs and ISEMFs with respect to those in non-treated cells. On the contrary H_2_O_2_ exposure significantly augmented the percentage of late apoptotic/necrotic cells starting 6 hrs after the stimulation (Table 1).

**Table 1:** Evaluation of cell death.

		HSC		ISEMF	
		A^+^	A^+^/PI^+^	A^+^	A^+^/PI^+^
**CTR**		2.3±1.2	6.5±2.4	3.2±1.5	7.5±2.1
**plasma**	**1 hr**	2.5±0.9	6.4±2.1	3.8±1.7	8.0±2.4
	**6 hrs**	5.7±3.2	6.2±2.7	6.1±3.2	7.8±2.5
	**12 hrs**	5.2±3.4	6.8±2.5	5.8±2.6	8.2±3.5
	**24 hrs**	4.4±1.3	10.4±3.0	5.1±2.8	11.3±2.8
	**48 hrs**	2.9±0.9	8.3±2.5	3.1±1.8	7.9±2.3
**H_2_O_2_**	**1 hr**	5.5±2.6	6.3±2.3	4.8±1.9	8.8±2.7
	**6 hrs**	15.3±5.3*	11.3±3.7	12.3±4.3	9.0±2.4
	**12 hrs**	16.9±4.8*	22.5±6.4*	12.4±2.1*	17.7±5.8
	**24 hrs**	16.8±7.4*	30.2±6.9*	13.5±3.9*	27.3±6.3*
	**48 hrs**	9.7±4.4*	27.7±5.2*	11.8±4.7	26.5±6.4*

Plasma-treated and H_2_O_2_ exposed cells were cultured for 1, 6, 12, 24, and 48 hrs. Cells were then stained with Annexin-V (A) and propidium iodide (PI) and evaluated by FACS analysis to assess cell death in 10,000 events. Data are reported as mean±SE of results collected in nine independent experiments, each performed in duplicate or triplicate.

* denotes P<0.05 *vs* not treated cells.

### Plasma treatment modulated PPAR-γ expression and cytokines production

**Figure 4:**
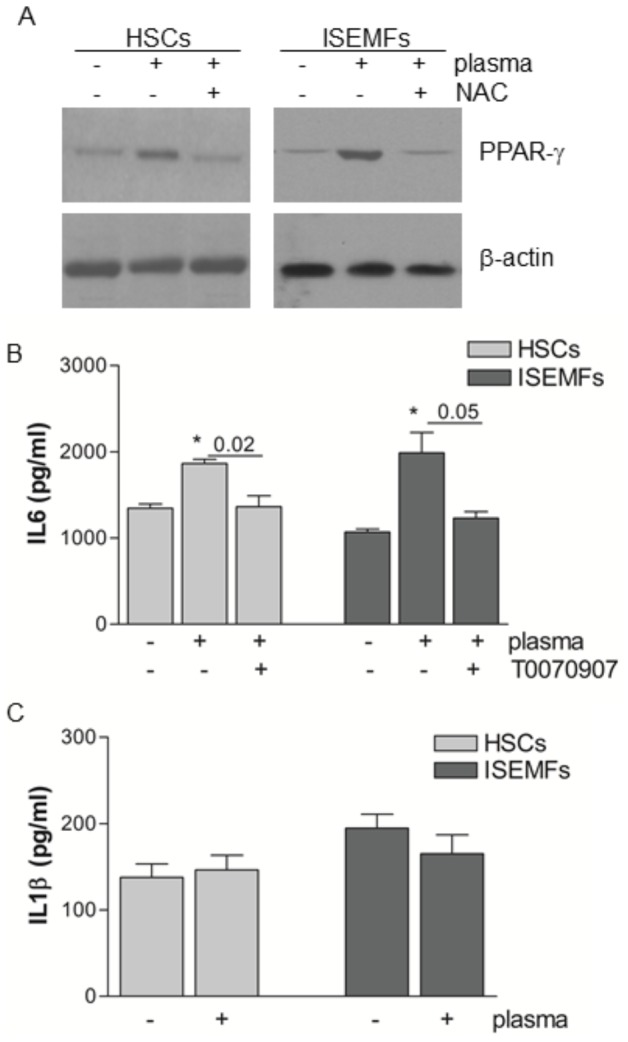
Intracellular signalling of plasma-induced ROS. Representative Western blot analysis of PPAR-γ in protein extracts from HSCs and ISEMFs treated with plasma and incubated for 4 hrs. As indicated, cells were pre-treated with NAC. Similar results were obtained in three independent experiments. β-actin was used as a loading control (panel **A**). Cells were treated for 30 min with 50 µM PPAR-γ receptor antagonist T0070907, exposed to plasma, and then incubated under standard conditions for 24 hrs. IL-6 (panel **B**) and IL-1β (panel **C**) were assessed in the conditioned medium by ELISA. Data are reported as mean±SE of results collected in nine independent experiments, each performed in triplicate. * denotes P<0.05 *vs* non treated cells.

Intracellular ROS act as transducing molecules in mammalian cells and engage different signaling pathways [32], [33]. PPAR-γ, in particular, is a transcription factor mainly involved in lipid metabolism and regulation of inflammation. Thus, PPAR-γ suppresses uncontrolled activation of HSCs and prevents inflammatory damage during colitis [34], [35]. As outlined in Figure 4A, plasma-generated ROS increased the expression of PPAR-γ in fibroblast-like primary cells 4 hrs after treatment, but pre-treatment of cells with the antioxidant agent NAC prevented the plasma-induced increase in PPAR-γ expression.

Plasma-induced activation of the PPAR-γ signaling pathway resulted in an increased production and secretion of IL-6, a cytokine involved in tissue regeneration [36]. Plasma-treated cells were cultured for 24 hrs at 37°C, and IL-6 expression in conditioned medium was assessed by ELISA. Plasma exposure increased IL-6 levels by 28% in the conditioned medium of HSCs and by 46% in that of ISEMFs. Cell pre-treatment with the PPAR-γ receptor antagonist T0070907 blunted the increase in IL-6 levels (Figure 4B). Plasma treatment did not increase the production of IL-1β (Figure 4C), TNF-α, or TGF-β (data not shown) in HSCs and ISEMFs.

### Plasma exposure promoted cell proliferation and migration

**Figure 5:**
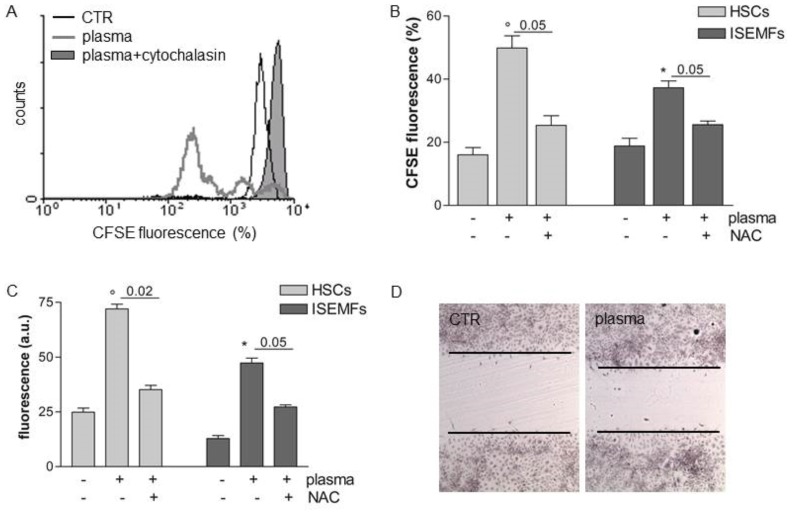
Proliferation and migration in plasma treated fibroblast-like cells. HSCs were labelled with 25 µM CFSE and incubated with NAC or 5 µg/ml cytochalasin B. Cells were then treated with plasma and incubated for 72 hrs. Panel **A** outlines representative FACS analysis of cellular proliferation assessed by evaluating the partition of CFSE. Similar results were obtained in nine independent experiments, each performed in triplicate. Panel **B** reports the percentage of proliferating fluorescent cells. Panel **C** reports the fluorescence relative to 6-CFDA in cells migrating to the bottom side of trans-well inserts (a.u. means arbitrary unit). Data are reported as mean±SE of results collected in nine independent experiments, each performed in triplicate. ° denotes P<0.02 *vs* non treated cells. * denotes P<0.05 *vs* not treated cells. Plasma treated ISEMFs were seeded onto glass coverslips. The cell monolayer was wounded using a plastic tip and 72 hrs later the cells were stained with haematoxylin and eosin. Images were observed and captured using a light transmission microscope connected to a camera (DMLB Leica, Panel **D**). Similar results were obtained in three independent experiments.

Cell proliferation was assessed evaluating the partitioning between daughter cells of CFSE probe-related fluorescence in cells cultured for 72 hrs, a condition established in our preliminary experiments. According to FACS analysis (Figure 5A), the fluorescence partitioning occurred in 15.9% of non- treated HSCs while plasma exposure stimulated proliferation in 49.8% of the cells. Cellular proliferation occurred in only 1.97% of HSCs pre-treated with cytochalasin B, an inhibitor of cell division (Figure 5A). Similar results were obtained in the ISEMFs (data not shown). The plasma-induced cell proliferation was secondary to intracellular ROS generation since NAC, a ROS scavenger, significantly abolished cellular division (P<0.05 *vs* plasma-treated cells, Figure 5B).

Besides increasing cellular proliferation, plasma treatment promoted migration of fibroblast-like primary cells. As outlined in Figure 5C, migration of plasma-exposed HSCs and ISEMFs labeled with 6-CFDA probe increased respectively by 2.67 fold and 1.62 fold as compare to non-treated cells. Again, cell migration was abolished by NAC pretreatment, demonstrating that plasma-induced cell migration is mediated by intracellular ROS formation. As a consequence of the increased cell proliferation and migration, plasma-treated ISEMFs also demonstrated marked ability in gap closure assay (Figure 5D).

## Discussion

Wound care and therapy have been drawing growing attention particularly in view of the increase in diseases associated with skin and inner lesions due to aging, diabetes and trauma. It is also known that atherosclerosis and defective nutrition can delay or jeopardize the repair process. The options available today to care for acute or chronic lesions are numerous but at the same time, several factors work against the repair/regeneration process [37]. According to the most up to date findings, normal wound repair after tissue injury follows a closely regulated sequence of events including the activation and proliferation of fibroblastic-like cells. In pathological situations the normal stages are altered and those processes continue inducing excessive accumulation of extracellular matrix [19], [38]. In view of these considerations, medical tools able to induce transient activation of fibroblast-like cells could be useful in promoting healing of lesions without inflammation and scarring [39]. In the present study we have demonstrated that non-thermal atmospheric pressure plasma ionizing helium gas mixed with air generates active chemical species that tune the activation of two fibroblast-like cell populations (human HSCs and ISEMFs) by inducing intracellular ROS.

For decades ROS have been considered accidental byproducts of oxygen metabolism having detrimental effects on cell survival. More recent investigations have however uncovered the dual nature of ROS and their role in sustaining normal biological functions [40], [41]. On the one hand, higher than normal ROS levels cause irreversible damage to cellular organelles, membrane, proteins and DNA; they have also been implicated in ageing, cancer, and neurodegenerative diseases [42], [43]. On the other, cells have evolved mechanisms such as antioxidant enzymes to modulate intracellular ROS levels [44]. Thus, at low/moderate concentrations ROS activate the intracellular signaling pathways resulting in production of soluble factors involved in cell growth and proliferation [41]. ROS generated in human HSCs and ISEMFs by plasma exposure were found here to be characterized by lower concentrations and shorter half-lives with respect to H_2_O_2_ induced ROS (Figure 2C). The transient increase in ROS levels spared cells early markers of apoptosis such as the decrease in the mitochondrial membrane potential (Figure 3A) and the externalization of phosphatidylserine (Table 1), an important recognition signal for macrophage activation and subsequent engagement of the inflammatory cascade [45]. On the contrary, higher levels of ROS induced in HSCs and ISEMFs by incubation with 20 µM H_2_O_2_ resulted in mitochondrial membrane damage and early cellular death (Table 1). For sure, cellular damage could be avoided by lower concentrations of H_2_O_2_. However, our unpublished dose-response experiments did not reveal a precise match between H_2_O_2_ concentrations and biological effects in primary cells putting forward the idea that generation of ROS by H_2_O_2_ incubation can not be modulated as easily as by plasma treatment.

Plasma generated ROS were found to increase PPARγ expression (Figure 4A), the transcription factor belonging to the nuclear hormone receptor superfamily. In recent years the pleiotropic effects of PPAR-γ agonists (e.g. regulation of energy metabolism, suppression of inflammatory activity, control of tissue remodeling) have become evident. PPAR-γ, nevertheless, engages different signaling pathways in a cell specific fashion mainly through the net balance of the soluble factors produced [33]–[35], [46]. In cultured human HSCs and ISEMFs, plasma triggered the production and secretion of IL-6, cell migration, and proliferation and prompted the closure of the gap, as was assessed by the *in vitro* wound healing model (Figures 4B and 5). Cellular migration is crucial during the wound healing process and is regulated by several intra- and extra-cellular mechanisms. Actin polymerization, for example, produces force that alters the cytoskeletal structures and leads to cell adhesion and spreading in the immediate vicinity of the wound [47]. The signals governing the uniform directionality of cell migration toward the repairing area are, however, not entirely understood [19]. Nishimura and colleagues reported that human keratinocytes move to the negative pole of the electric fields generated near wounds in mammalian skin [48]. The electrically directed movement, known as galvanotaxis, is not, however, shared by all cells involved in the wound healing process [49]. Findings from the present study demonstrated that migration and proliferation in the plasma-exposed HSCs and ISEMFs are ROS dependent since biological effects were significantly reduced by pre-treatment of cells with the NAC antioxidant agent (Figure 5B and C). Indeed, a growing body of studies published by Lindequist U. and co-workers has clearly demonstrated that in the non-tumorigenic human keratinocyte cell line HaCaT plasma-generated ROS influences the expression of the surface pattern receptors (e.g. integrins) mainly involved in cellular protrusion [10], [17], [50], [51]. It is well known, nevertheless, that plasma components (such as radiations, ionized nitrogen or ROS), plasma power, the distance from the sample and exposure time affect cells differently. The numerous parameters influencing the performance of various plasmas could explain the differences in cell death and cell migration that have been outlined in other works [10], [52]. As clearly described in our own study, even the levels of intracellular ROS are important in tuning the cellular effects (Figure 2C and Table 1, plasma *vs* H_2_O_2_ treatment). Volotskova O. and colleagues, moreover, reported that following 100 s of treatment the reduction in fibroblast migration was affected by the distance from the treated area. Cells had the slowest migration rate in the plasma treated zone while those outside the treated area (5–8 mm away) migrated at the same rates as the untreated cells [52]. Even if the authors did not investigate intracellular signaling, it seems that their plasma jet did not generate reactive diffusible ROS.

Mitochondria, peroxisome, cytoplasmic proteins and lipid layers constitute the main sources of ROS in eukaryotic cells. Our findings demonstrated that 2 min of treatment with helium generated plasma does not alter the mitochondrial membrane potential in fibroblasts thus excluding these organelles as source of ROS (Figure 3A). Administration of cytoplasmic free radical scavengers, on the other hand, significantly dampened the plasma-induced generation of ROS (Figure 3B) suggesting that cytoplasmic proteins and membrane lipids are the eligible substrate for ROS formation after plasma exposure. Indeed, plasma treatment induced early transient lipid peroxidation in the cytoplasmic compartment (Figure 3C). The rapid drop in plasma-induced ROS generation prompted us to hypothesize that just as other human primary cells [3], HSCs and ISEMFs are endowed with an efficient armamentarium of scavenger enzymes to contain the oxidative burst and to avoid mitochondrial involvement thus preserving ATP production. ROS kinetics elicited in primary cells by plasma treatment significantly differed from those described in tumor cells where hypoxia and nutrient deprivation usually result in mitochondrial dysfunctions boosting ROS production and accounting for an increased rate of death of tumor cells in response to oxidative stress (our unpublished data) [6], [53].

Our results have clearly demonstrated that helium generated plasma treatment induces proliferation and migration of human fibroblast-like primary cells mainly through intracellular ROS formation. Since the threshold of ROS is crucial in cellular signaling and their levels could be modulated in the intracellular compartment by non-thermal atmospheric pressure plasma, it represents a promising tool in the control of fibroblast activation. As outlined in Figure 2B, differences of only fractions of a millimeter in the distance between the plasma needle and the cellular medium affect ROS generation. Moreover, as recently reported, the composition of the medium embedding the samples greatly influences ROS generation in plasma treated cells [51]. Taken together, these data demonstrate that the effects of plasma can be modulated depending on the nature of the sample and the aim of the treatment. At the same, however, it is by now unquestionable that *in vivo* plasma treatment requires availability of accurate standardized devices as well as in depth knowledge about the biological significance of ROS formation in different cell populations. While more studies are required to investigate DNA damage induced by low transient levels of ROS in primary cells, additional clinical trials investigating the tolerability of plasma treatments [1] and further developments in the technology of atmospheric cold plasma generated using small needles will hopefully pave the way for endoscopic applications in the event of liver and gut lesions.
